# Blockchain and digital finance

**DOI:** 10.1186/s40854-022-00420-y

**Published:** 2022-11-28

**Authors:** Wei Xu, Daning Hu, Karl Reiner Lang, J. Leon Zhao

**Affiliations:** 1 Renmin University of China, Beijing, China; 2 Southern University of Science and Technology, Shenzhen, China; 3 City University of New York, New York City, USA; 4 The Chinese University of Hong Kong, Shenzhen, China

Blockchain technology and its applications in various business domains have attracted great attention from researchers and practitioners in recent years. Finance, which is arguably the most promising and well-known application domain, has been significantly transformed into digital finance by various novel and open technological and business innovations rooted in blockchain technology, such as decentralized finance and cryptocurrency. Digital finance innovations like digital payments, crowdfunding, supply chain finance, and robo-advising have made significant progress. The main goal of this special issue is to deepen and broaden our understanding of the impacts, values, and challenges brought by blockchain technology and digital finance.

Blockchain technology is a distributed ledger introduced for cryptocurrency in 2008 by Satoshi Nakamoto. This technology is poised to become one of the most exciting new inventions after the Internet as it resolves the trust issue efficiently via network computing (Zhao et al. 2016). The article in the Economist Magazine on October 31, 2015 suggested blockchain as the trust machine that would shape how businesses are operated and how transactions are executed. In Finance, it not only changes the way how financial information can be stored and processed securely and efficiently, but can also transform the principles and processes embedded in traditional centralized financial institutions. The articles included in this special issue show how blockchain technology can be applied in novel ways for supporting the transformation of business processes and operations in digital Finance.

A large number of submissions were received in response to the call for papers on the theme of blockchain and digital finance. After an extensive review process, 15 of them were included in this 36th volume of Financial Innovation (FIN), Volume 8, No. 6 (2022). Next, we summarize the key outcomes of these articles and then identify an agenda for future research that has emanated from them.

## A summary of the special issue papers

Since cryptocurrency is the earliest and most established application of blockchain technology in digital finance area, most papers in this special issue are related to this topic. These papers can mainly be categorized into four sub themes. The first one is mainly regarding to the price prediction of cryptocurrency and crypto-related assets. Gurrib et al. (2022) find that Fibonacci retracement, a popular technical analysis indicator, captures energy stock prices better than energy cryptocurrencies. Critien et al. (2022) build a model to predict the direction and the magnitude of Bitcoin price changes by analyzing the sentiment and data volume of tweets. Li et al. (2022) propose a deep-learning model for forecasting the daily price changes in the Bitcoin market and algorithmic trading. The proposed model obtains higher investment returns than benchmark models in a trading simulation.

The second sub-theme is mainly about investor behavior and market phenomena in cryptocurrency markets. Blasco et al. (2022) study the herding effect among exchanges before the Bitcoin futures expiration date. Haykir and Yagli (2022) investigate financial bubbles in the cryptocurrency market during the COVID-19 pandemic. They suggest that bubbles are common in cryptocurrency markets, which is inconsistent with the efficient market hypothesis. Fratrič et al. (2022) design an agent-based model to reproduce Bitcoin market participants’ behaviors during the time of an alleged Bitcoin price manipulation. Hasan et al. (2022) examine the dynamics of liquidity connectedness in the cryptocurrency market. They report that there is a moderate liquidity connectedness among six major cryptocurrencies, with Bitcoin and Litecoin playing a significant role concerning the magnitude of connectedness. Moreover, Cui and Maghyereh (2022) study the higher-order moment co-movements and risk connectedness among cryptocurrencies before and during the COVID-19 pandemic. Lorenzo and Arroyo (2022) describe, summarize, and segment the main trends of the cryptocurrency market in 2018. Ma and Tanizaki (2022) investigate the phenomenon of price clustering in the Bitcoin market that is denominated in the Japanese yen. The last two digits of Bitcoin price are found to cluster at the numbers that end with ‘00’.

The third group provides systematic literature reviews of cryptocurrency related studies. García-Corral et al. (2022) review 1419 cryptocurrency related articles published between 2010 and 2019, and discuss the evolution of blockchain technology used in cryptocurrency. Fang et al. (2022) provide a comprehensive survey of 146 research papers on cryptocurrency trading and identify opportunities in cryptocurrency trading research.

The fourth group studies cryptocurrency from other novel perspectives. Shibano and Mogi (2022) discuss two cases in which issuers can stimulate cryptocurrencies to attain a monetary function. Minutolo et al. (2022) find that the impacts of COVID-19 outbreak on the price change and trading volume of cryptocurrencies vary by trading currencies and regions. Campino et al. (2022) study the factors that affect the success of initial coin offering (ICO) projects, including a well-structured and informative whitepaper, the proximity to the markets with high availability of financial and human capital, social media in ICO projects, etc.

## Implications for future research

The first group of papers in this special issue on cryptocurrency price prediction considers features such as the sentiment and volume of tweets from market participants, decomposed frequency modes, and technical analysis indicators. Other novel features in price prediction that can be further explored may include sentiment extracted from social media videos (Fang et al. 2022) and features from external financial environments (Li et al. 2022). Moreover, from the design science perspective, a related future research direction is to develop a more user-friendly decision support system tailored for blockchain-based technological ecosystems with accurate price predictions for investors.

The second group of studies relating to cryptocurrency investor behavior and market phenomena suggests several lines of future research. For example, to extend the study of Hasan et al. (2022), future research may explore the determinants of liquidity connectedness in the cryptocurrency market. To extend Cui and Maghyereh (2022), future research can investigate the higher-order moment portfolio optimizations of cryptocurrencies. Another promising research direction is to examine the extreme risk connectedness among cryptocurrencies from a time–frequency domain perspective. To further understand bubbles in the cryptocurrency market (e.g., Haykir and Yagli 2022), one may consider to develop models tailored for cryptocurrency market rather than traditional financial markets. Regarding bubbles during the COVID-19 pandemic, future studies couldexamine the monetary policy changes in different pandemic phases and compare the results with those of Haykir and Yagli (2022).

There are several other lines of research that may need to be further explored. First, whether the results obtained in Shibano and Mogi (2022) can be applied in the real world needs to be examined and validated. Second, future research may extend the analysis results on cryptocurrency by Minutolo et al. (2022) to other crypto asset classes, such as non-fungible tokens (NFTs). It would be interesting to see whether the effect of the COVID-19 outbreak on major cryptocurrencies is also similar for other crypto assets. Moreover, considering the characteristics of cryptocurrency, the correlation between cryptocurrency and other crypto assets (in extreme conditions) also needs to be further studied (Fang et al. 2022). Finally, certain technical factors like the whitepaper, are found to significantly influence ICO project success (Campino et al. 2022). It would also be interesting to analyze the social and economic factors within the ICO project, such as social relationships among project members and community governance.

This special issue mainly focused on blockchain technology and its applications in digital finance, most prominently- cryptocurrencies, from various perspectives. The articles included provide many important implications. While blockchain technology has enabled cryptocurrency, its widespread adoption in digital finance has led to many research opportunities in other important blockchain applications, such as Decentralized Finance and NFTs.
